# The Central Role of the Gut Microbiota in Chronic Inflammatory Diseases

**DOI:** 10.1155/2014/689492

**Published:** 2014-09-18

**Authors:** Caroline Marcantonio Ferreira, Angélica Thomaz Vieira, Marco Aurelio Ramirez Vinolo, Fernando A. Oliveira, Rui Curi, Flaviano dos Santos Martins

**Affiliations:** ^1^Department of Pharmacology, Institute of Biomedical Sciences, ICB-1, Sao Paulo University, 05508-000 São Paulo, SP, Brazil; ^2^Department of Genetics, Institute of Biological Sciences, Federal University of Minas Gerais, 31270-901 Belo Horizonte, MG, Brazil; ^3^Department of Genetics, Evolution and Bioagents, Institute of Biology, University of Campinas, 13083-970 Campinas, SP, Brazil; ^4^Department of Biological Sciences, Section of Physiology and Pharmacology, Federal University of Sao Paulo, 09913-030 Diadema, SP, Brazil; ^5^Department of Physiology and Biophysics, Institute of Biomedical Sciences, ICB-1, Sao Paulo University, 05508-000 São Paulo, SP, Brazil; ^6^Department of Microbiology, Institute of Biological Sciences, Federal University of Minas Gerais, 31270-901 Belo Horizonte, MG, Brazil

## Abstract

The commensal microbiota is in constant interaction with the immune system, teaching immune cells to respond to antigens. Studies in mice have demonstrated that manipulation of the intestinal microbiota alters host immune cell homeostasis. Additionally, metagenomic-sequencing analysis has revealed alterations in intestinal microbiota in patients suffering from inflammatory bowel disease, asthma, and obesity. Perturbations in the microbiota composition result in a deficient immune response and impaired tolerance to commensal microorganisms. Due to altered microbiota composition which is associated to some inflammatory diseases, several strategies, such as the administration of probiotics, diet, and antibiotic usage, have been utilized to prevent or ameliorate chronic inflammatory diseases. The purpose of this review is to present and discuss recent evidence showing that the gut microbiota controls immune system function and onset, development, and resolution of some common inflammatory diseases.

## 1. Introduction

Commensal microbiota consists of many microorganisms that cover all host mucosal surfaces, but most reside in the gastrointestinal tract, which is the subject of this review. Amazingly, although the human body is composed of approximately 100 trillion cells, only 10 trillion are human cells while 90 trillion are microbes. The genes of these microorganisms form our metagenome, known as our second genome [1]. Thus, it is not surprising that this large arsenal of gene products has a relevant role in body homeostasis [2, 3]. The relationship between the gut microbiota and its host plays a key role in immune system maturation, food digestion, drug metabolism, detoxification, vitamin production, and prevention of pathogenic bacteria adhesion [4]. One of the most important roles of the microbiota is the maturation of the immune system in the postnatal period. The first appearance of adaptive immunity in humans coincides with acquisition of a complex diet and microbiota, which suggests that mucosal immunity in the intestines has evolved to tolerate diverse microbes and food antigens.

Colonization of the gastrointestinal tract begins after birth, despite the fact that some researchers have discovered a small community of bacteria living in the placenta [5]. However, there is no convincing evidence demonstrating that such bacteria normally reach the fetus through the placenta. It is known that colonization initiates from maternally acquired bacteria during birth [6] and breastfeeding and continues throughout our life [7–9]. Over the lifetime of the individual, or at least until stabilization of colonizing microbiota in adulthood, there is a change in the profile of the predominant phyla in the gastrointestinal tract, migrating from a community dominated by Actinobacteria and Proteobacteria to one dominated by Firmicutes and Bacteroidetes [10]. The metagenome of an infant gut is characterized by an enrichment of genes required for the breakdown of simple sugars, such as lactose and galactose, while the weaned infant microbiota is enriched in genes for polysaccharide breakdown and vitamin production [11, 12]. Most bacterial species in the human and mouse gut belong to the phyla Bacteroidetes and Firmicutes, but less abundant bacterial phyla, such as Actinobacteria, Proteobacteria, and Verrucomicrobia, as well as methanogenic archaea, mainly* Methanobrevibacter smithii*, are also present [13, 14].

The composition of the microbiota is influenced by environmental factors such as diet, antibiotic therapy, and environmental exposure to microorganisms. Additionally, it can vary according to sex, age, and geographical origin of the individual [15]. An overgrowth of pathogenic microbial colonies causes an imbalance known as dysbiosis. Antibiotic therapy, alcohol misuse, and inappropriate diet are factors that can lead to dysbiosis [16–18].

The normal relationship between the gut microbiota and the immune system is established by bacteria, cells, and receptors of both the innate and adaptive immune systems. Microbes are held in the intestinal lumen through the combined efforts of the epithelial barrier, mucus layer, antimicrobial peptides, and antibodies. Controlling the intestine's metabolic products is also important for the maintenance of a mutually beneficial relationship between the microbiota and the immune system. When this connection is broken and fails to resolve itself, an inflammatory response is initiated.

Here, we review the mechanisms by which the gut microbiota contributes to the development of asthma, bowel disease, and obesity, highlighting the regulatory role of the gut microbiota in immune system function.

## 2. Mechanisms Linking the Microbiota and Its Products to the Immune System

Recently, several studies have shown possible links between the gut microbiota and the immune system. Here, we summarize some of the “sensors” that are involved in this interaction and describe related pathological conditions.

Innate immune cells such as macrophages, neutrophils, and dendritic cells, as well as other cell types including epithelial cells, which form the interface between the body and the external environment and are in close contact with the microbiota, express several membrane and intracellular proteins that sense microbial molecules. Examples of these sensors include pattern-recognition receptors such as Toll-like receptors (TLRs), C-type lectin, nucleotide oligomerization domain (NOD) receptors (NLRs), and retinoic acid inducible gene (RIG)-I-like receptors (RLRs), which are activated by microbial molecules including flagellin, lipopolysaccharide, lipoteichoic acid, peptidoglycans, N-acetylglucosamine, and double stranded-RNA. Considering the types of ligand that activate these receptors, it is not surprising that some participate in the microbiotic regulation of the immune system and serve as regulators themselves. These receptors also play a role in shaping the microbiota. For example, in the absence of TLR5, a receptor activated by bacterial flagellin, mice present changes in microbiota composition that have been associated with the development of metabolic syndrome in these animals [19].

NLR proteins are expressed in a wide variety of both immune and nonimmune cells and detect microbial and endogenous signals released from these cells. These proteins consist of three domains: a central nucleotide-binding domain termed NACHT (referred to as the NOD domain) and both amino- and carboxy-termini consisting of leucine rich repeats (LRR domains). These latter two domains are important, respectively, for interaction with other proteins that initiate a signaling cascade and for recognition of molecules that activate a family of receptors comprising 22 different human proteins. These proteins are classified based on their N-terminal domain, which includes a caspase recruitment domain (CARD) on Nod1, Nod2, and NLRC3, 4 and 5; a pyrin domain (PYD) on NLRP1-14; an acidic transactivating domain on NLRA; or a baculovirus inhibitor repeat (BIR) on NAIP [20]. Several studies have shown that changes in the expression of these intracellular sensors lead to modifications in the composition (both qualitative and quantitative) of the microbiota and the immune system and have been associated with the development of conditions including colitis, bacterial infection, obesity, and insulin resistance [21].

Another class of sensors that detects molecules derived from the microbiota is the G protein-coupled receptors (GPCRs). These receptors will be discussed in detail below. At least three GPCRs have been identified that bind to short chain fatty acids (SCFAs) produced by gut bacteria: GPR41, GPR43, and GPR109A. GPR41 (i.e., FFAR3) and GPR43 (i.e., FFAR2) are both highly expressed on immune cells such as polymorphonuclear cells and macrophages [22]. Additionally, other cells and tissues including adipose tissue, enteroendocrine cells and the cells of the sympathetic nervous system have also been shown to express these receptors and to mediate some of their biological effects [23]. G protein-coupled receptors are activated by SCFA; butyrate binds to GPR41 with high affinity, but acetate and propionate have a greater affinity for GPR43 [24–26]. Some of the effects associated with SCFA depend on the activation of GPR43 and include reactive oxygen species (ROS) production and neutrophil chemotaxis. More recently, it has been shown that via this receptor, SCFA modulates the number of T regulatory cells (Tregs) in the colon, an effect that will be further described in last section of this review. GPR41 activation has been associated with regulation of metabolism and energy expenditure.

GPR43 is reported to activate both Gi/o and Gq, while GPR41 signals via Gi/o only. Both receptors induce intracellular calcium mobilization and inhibit cAMP accumulation. The MAPKs ERK1/2, JNK, and p38 are activated by SCFAs through binding to the GPCRs [27]. GPR41 and GPR43 activation of ERK1/2 is dependent on Gi/o, because the inhibition of this G protein by the pertussis toxin abolishes the stimulatory effect of SCFAs on this pathway in cells expressing only the GPR41 and reduces it in more than 50% in cells expressing GPR43 alone [27]. In neutrophils, ERK1/2, p38, and PKB are activated by SCFAs through a pertussis toxin sensitive pathway and are important for GPR43-dependent chemotaxis. A recent study has demonstrated a chemotactic of SCFAs through a mechanism involving PI3K*γ* and the small G protein Rac2 [23]. Recently, it has also been shown that SCFAs induce chemokine and cytokine expression in colonic epithelial cells in a GPR41- and GPR43-dependent manner. In this study, the authors demonstrate the involvement of Gi/o, ERK, p38, and the transcription factor ATF2 in this SCFA-induced expression [28].

GPR109A, also known as hydroxy-carboxylic acid 2 receptor or HM74a, is a receptor for nicotinate. Additionally, this protein binds to the ketone body *β*-D-hydroxybutyrate and to the SCFA butyrate [29, 30]. This receptor is expressed on hematopoietic-derived cells, white and brown adipocytes, keratinocytes, colonocytes, and hepatocytes [29, 30].

## 3. Asthma

Asthma is a chronic airway disease characterized by excessive contraction of airway smooth muscle (termed airway hyperresponsiveness or AHR), exacerbated mucus production, eosinophilia, and elevated Th2 cytokine production [31]. Asthma affects approximately 300 million people worldwide, and it is estimated that in 2025, more than 100 million people will be diagnosed with this pathology [32, 33]. Current treatment is based on anti-inflammatory therapies, which do not cure asthma. Furthermore, AHR may persist even in the absence of inflammation. The treatment of asthma is complex because there are many asthma phenotypes, and its effectiveness depends on environmental and genetic factors [34]. Asthma prevalence is increasing in Western countries due to lifestyle modifications including excessive hygiene (i.e., little exposure to microbes) and use of antibiotics and a high-fat diet [35–40]. Epidemiological studies have shown that exposure to microbes early in life is a critical factor in the induction of allergic diseases, leading to the development of the hygiene hypothesis [35–39]. Briefly, this theory proposes that excessive cleaning and reduced pathogen exposure leads to an inadequate immune response [41]. Likewise, the use of antibiotics early in life is also associated with allergic sensitization and AHR [42]. Thus, exposure to microbes early on has a great influence on immune function later in life. Moreover, the intestinal microbiota, our largest collection of microorganisms, modulates the pathophysiological processes of asthma. Several groups have noted that the hygiene hypothesis should be rewritten to include the role of the intestinal microbiota and thus renamed as the “microflora hypothesis.” The “microflora hypothesis,” initially discussed by Noverr and Huffnagle [43], postulates that perturbations in the gastrointestinal microbiota, resulting from reduced microbial exposure due to changes in diet and antibiotic use [44], lead to an underdeveloped microbiota. This “immature” microbiota delays proper maturation of the immune system. The sequence of events that promotes the development of immunological tolerance is disrupted, leading to allergic hypersensitivity [43].

Epidemiologic studies have identified associations between alterations in the composition of gut bacterial communities and the development of allergies [45, 46]. Children with asthma have a different intestinal microbiota compared to nonasthmatic children. Asthmatic children have a high prevalence of certain species of* Clostridium difficile* (bacterium with pathogenic characteristics) and low* Bifidobacterium* (nonpathogenic bacteria) in their intestinal microbiota [45, 47]. Clinical trials have indicated that feeding* Lactobacillus rhamnosus* GG and* Lactobacillus fermentum* to mothers in the prenatal and early postnatal periods may be effective in the treatment and prevention of early atopic disease in children [47, 48].

Studies in animal models have also shown that gut bacteria modulate experimental asthma [22, 49]. Researchers have employed three main strategies to interfere with gut colonization and showed its effects beyond the local gut immune response. These strategies include maintaining germ-free (GF) mice (devoid of microbiota) in a sterile environment, microbiota depletion/perturbation by antibiotic therapy, and alteration of the microbiota composition through modification of the host's diet.

**Figure 1:**
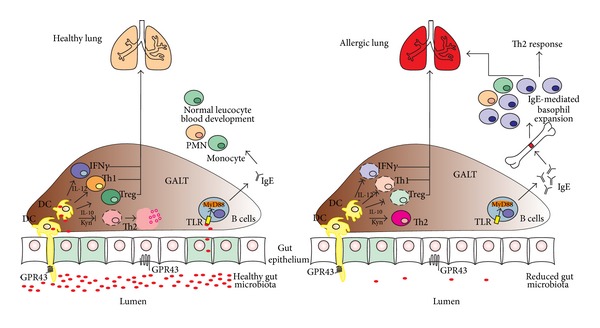
Schematic representation of the pulmonary allergic response induced by gastrointestinal (GI) immune cells and two microbiota-related conditions (a healthy gut microbiota and a reduced gut microbiota following antibiotic treatment). Microbes in the intestines are sampled by Toll-like receptors (TLRs) on DCs either directly in the lumen or in the gut-associated lymphoid tissue (GALT). In the healthy gut microbiota, polymorphonuclear development (PMN) is normal and DCs become regulatory DCs (DCr) that promote development of Tregs and/or Th1 cells and natural killer (NK) cells. These NK cells inhibit Th2 inflammation. Antibiotic treatment kills a large proportion of healthy microbiota, leading to a reduced gut microbiota and an inflammatory environment without DCs, Th1 cells or NK cells. In this environment, an unhealthy microbiota elevates serum immunoglobulin E (IgE) levels, increases circulating basophil populations, and exacerbates basophil-mediated Th2 responses (adapted from Forsythe [110]).

The mechanisms by which the innate immune system recognizes the commensal-derived signal that regulates Th2 inflammation is currently being studied. Dendritic cells (DCs), basophiles, and invariant natural killer T (iNKT) cells are part of this mechanism. DCs are the primary antigen-presenting cells responsible for the antigen-specific activation of naive T cells. Microbes in the intestine are sampled by DCs either directly from the lumen or through the gut-associated lymphoid tissue (GALT). A combination of signals from microbes results in phenotypic changes in the DCs, which leads to the differentiation of Th1, Th2, and Treg cells (Figure 1). One phenotypic change is the increased production of IL-10 by these cells. DCs expressing high levels of IL-10 drive the generation of CD4+FOXP3 Tregs and the establishment of tolerance. Tolerance can be established by the activation of Th1 and Treg cells. This regulatory mechanism plays a key role in the immunoregulatory action of many probiotics. In this way, the intestinal microbiota may induce Treg cells in the GALT that then spread to the airways in response to allergen exposure. This idea is supported by the finding that oral treatment with* Lactobacillus reuteri* results in an increase in Treg cells in the draining lymph nodes of the lung. Additionally,* L. rhamnosus GG* has been shown to reduce the murine allergic airway response through associated increases in FOXP3T cells only when bacteria are administered in the neonatal period [50, 51]. The generation of Treg cells is only one mechanism; other mechanisms may also account for the effects of microbiota in immune regulation, as discussed below.

Germ-free mice exhibited an exaggerated number of airway eosinophils, increased production of Th2 cytokines, elevated immunoglobulin E (IgE) production and an altered number and phenotype of DCs when sensitized and challenged with ovalbumin (OVA). This phenotype was abolished by recolonization of germ-free mice with the complex commensal flora of specific pathogen-free mice [52]. Interestingly, Tregs were unaffected in GF mice, although the number of basophils was increased. Moreover, depletion or deletion of bacterial communities was associated with elevated serum IgE concentrations, an increased circulating basophil population, exaggerated Th2 cells responses, and allergic inflammation [53] (Figure 1). Additionally, the exaggerated Th2 response was reduced upon depletion of basophils. Thus, basophils are an important link between the gut microbiota and allergic inflammation. Recently, investigators have discovered a mechanism by which commensal bacteria regulate basophil functions, interfering with the susceptibility of the Th2 immune response. They found that treatment with oral antibiotics increased serum IgE concentrations by increasing the level of circulating basophils and inducing an exaggerated Th2 inflammation. B cell-intrinsic expression of MyD88 is an important step in increasing serum IgE and basophil levels. When expression of MyD88 is blocked by a healthy microbiota, there is no development of allergic airway inflammation (Figure 1) [52].

Treg cells and basophils are not the only cell types affected by microbiota in mouse models. GF mice contain an increased number of iNKT cells compared to specific pathogen free (SPF) mice [49]. iNKT cells secrete abundant levels of IL-4, IL-12, and IFN-*γ* upon activation, resulting in increased susceptibility to allergic inflammation. Moreover, greater Th2-mediated airway inflammation was observed in GF mice than in SPF mice when mice were sensitized with OVA. Asthma in GF mice was CD1-d dependent, because depletion of these cells decreased allergic inflammation. Interestingly, researchers also observed that colonization of neonatal, but not adult, GF mice with conventional microbiota protected the animals from mucosal iNKT accumulation and asthma [49]. Thus, microbial contact early in life is critical for the establishment of mucosal iNKT cell tolerance to antigens in exposed airways.

In addition to innate immune cells, other elements related to microbiota may be important in the regulation of Th2-mediated airway inflammation. SCFAs are the major end products of bacterial metabolism in the human large intestine. The fermentation of complex plant polysaccharides leads to the production of SCFAs such as propionate, butyrate, and acetate. As described above, SCFAs have been reported to show anti-inflammatory properties such as leukocyte recruitment, leukocyte chemotaxis, and chemokine production [54, 55]. Animals deficient in a receptor coupled to GPR43 that binds to SCFAs, including acetate, have an exaggerated inflammatory response in models of colitis, arthritis, and asthma. OVA-sensitized GPR43 KO mice have a greater inflammatory infiltrate in the airways and lung tissue compared to littermate mice [22]. Moreover, Trompette et al. [56] found that fermentable dietary fiber content changed the composition of mouse gut and lung microbiota by altering the ratio of Firmicutes to Bacteroidetes bacteria, which consequently increased the concentration of SCFAs, specifically propionate. Mice fed a high-fiber diet were protected against allergic inflammation in the lungs by increased DC phagocytic function, although the DCs also displayed an impaired ability to mediate Th2 airway inflammation. Altogether, these studies suggest that SCFAs are important in controlling allergic pulmonary inflammation. However, there are only a few studies showing SCFA modulation of immune system function. The mechanism by which SCFA reduces AHR remains unknown. All SCFAs have the same effect on airway inflammation and lung function. The question, of which microbiota is more important for immunological responses in the airways, lung microbiota or gut microbiota, remains unanswered.

As a whole, the gut microbiota has a significant effect on airway immunity. Therefore, it is relevant to consider the composition of the host microbiota with the same level of importance as genetic polymorphisms and environmental factors when diagnosing and treating asthma.

## 4. Inflammatory Bowel Disease

The incidences of inflammatory bowel disease have risen rapidly over the last several years. Crohn's disease and ulcerative colitis are the main inflammatory bowel diseases (IBDs) and are characterized by a chronic and exacerbated inflammation of the intestinal mucosa [57]. In addition to genetics, several factors contribute to the high incidence of IBDs such as lifestyle and the intestinal microbiota. Commensal microbiota plays an important role in the pathogenesis of inflammatory bowel disease [58, 59] because experimental colitis has been successfully treated with an antibacterial agent [60] and antibodies against microbial antigens in IBD patients [61]. In experiments, GF mice were more susceptible to colitis induced by dextran sodium sulfate (DSS) [22]. Recolonization of GF mice with feces from conventional mice reversed this phenotype, showing that microbiota plays a beneficial role in colitis [22]. It is clear that dysbiosis results in a lack of immune regulation and breakdown of tolerance to commensal microorganisms. Dysbiosis allows outgrowth of more pathogenic microorganisms and promotion of the exacerbated inflammation underlying IBD [62]. Abnormal gut colonization has been observed in subsets of Crohn's disease and ulcerative colitis patients [63]. Patients with IBD, compared to healthy controls, have fewer bacteria with anti-inflammatory properties and/or more bacteria with proinflammatory properties [64]. In addition, an abnormal microbiota can cause IBD by expansion of colitogenic strains that initiate development of colitis [65]. The molecular mechanisms involved in the indirect effects of the microbiota on the host intestine in inflammatory bowel disease are described below.

**Figure 2:**
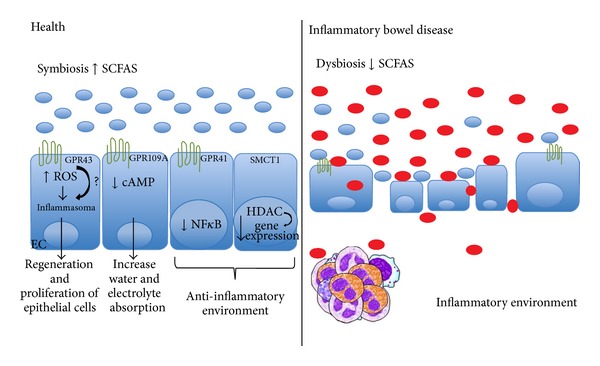
Schematic representation of a complex mucosal immune system composed of epithelial and hematopoietic cells that are able to react to pathogenic insults. Development of IBD occurs mainly when epithelial cells are damaged and/or the intestinal microbiota composition is not healthy.

The host develops a complex mucosal immune system composed of epithelial and hematopoietic cells in order to avoid ongoing inflammatory reactions to the microbiota and preserve its ability to react to pathogenic insults. When such interactions are perturbed, an exacerbated inflammation occurs, leading to the development of IBD [55]. Recent findings have focused on the molecular mechanisms involved in the interaction between the gut microbiota and epithelium cells [66–69] (Figure 2). The intestinal epithelium is more than a single layer of cells working as a physical barrier; it has developed mechanisms to protect itself from uncontrolled inflammatory responses and to prevent bacterial dissemination to other organs. The epithelial responses against the gut microbiota highlight the importance of a self-limiting or noninflammatory cellular immune response scenario in the antigen-rich intestinal environment. The reestablishment of intestinal barrier integrity regulates the inflammatory response in IBD [65, 68, 70]. GF mice recolonized with gut microbiota have shown a marked reduction in inflammation. The exacerbated response in colitis was related to a lack of bacterial colonization of the gut that provides beneficial effects in IBD [22]. Bacteria likely protect against IBD by directly or indirectly enhancing the intestinal environment via increased production of molecules such as SCFA by beneficial bacteria. SCFAs, mainly acetate, propionate, and butyrate, which are produced by bacteria of the Bacteroidetes and Firmicutes phyla after fermentation of dietary fiber, show anti-inflammatory properties in IBD [22, 71, 72]. Patients with colitis and/or Crohn's disease have reduced levels of these bacteria in the colon [63]. The SCFAs carry out many functions in the gut such as serving as fuel for the intestinal epithelium cell, regulating gut epithelium cell proliferation, differentiation, and gene expression, and initiating anti-inflammatory effects on intestinal mucosa [22, 73–76].

Butyrate elicits biological effects on intestinal epithelial cells by binding to GPR109A, a G protein-coupled receptor, which is highly expressed in the colon [29]. Activation of the GPR109A receptor by butyrate leads to a decrease in intracellular levels of cAMP and this reduction controls electrolyte and water absorption to reduce the incidence of diarrhea in IBD [77]. SLC5A8, known as SMCT1 (sodium-coupled monocarboxylate transporter 1), is a butyrate transporter in a Na^+^-dependent electrogenic process and is highly expressed in the colon. Butyrate has the ability to influence gene expression in the colon through histone deacetylase (HDAC) inhibition [78]. Interestingly, the expression of SLC5A8 and GPR109A in the gut is influenced by bacteria colonization. In the intestines of GF mice, the absence of the microbiota and consequently the absence of SCFAs leads to marked suppression of SLC5A8 and GPR109A expression [79]. In contrast, colonization of GF mice leads to expression of these genes to levels comparable to those of normal conventional mice [80]. Thus, lack of expression of these genes in GF mice could render them more susceptible to developing experimental colitis and Crohn's disease. Reduction in the intracellular availability of butyrate in colonocytes may decrease its protective effects in IBD patients. Butyrate, through a different mechanism, has also been shown to be protective against colonic inflammation and colon cancer [30]. Gpr109A, activated by butyrate, suppresses intestinal inflammation by (1) induction of IL-18 secretion in colonic epithelium, consequently inducing epithelium homeostasis, and (2) promoting an anti-inflammatory response in colonic macrophages and DCs that induce differentiation of Tregs. CD4+Foxp3+ T regulatory cells are indispensable for maintaining immune tolerance and are also an emerging therapeutic target for IBD. Recent studies have demonstrated that the metabolic products of certain bacterial strains in the intestines attenuated disease in animal models of colitis by inducing Treg proliferation [81–83]. These bacteria also promoted their peripherical generation by inducing T cell differentiation to Tregs through the generation of a TGF-*β*-rich environment [84].

The effects of SCFA may also result from its binding to GPR41 and GPR43. Indeed, GPR41-deficient mice have a higher susceptibility to experimental colitis, and this phenotype is associated with greater activation of NF-*κ*B (Nuclear Factor kappa B). Activation of NF-*κ*B induces expression of genes responsible for the production of proinflammatory cytokines such as TNF and IL-8 that contribute to the pathogenesis of IBD [85]. However, butyrate displays an anti-inflammatory effect by decreasing expression of proinflammatory cytokines via inhibition of NF-*κ*B activation [86, 87]. A marked anti-inflammatory effect was observed by acetate as well. The effects of acetate have been demonstrated by Maslowski et al. to be due, in part, by the activation of GPR43 [22]. GPR43-deficient mice exhibit aggravated inflammation related to exacerbated production of inflammatory mediators and increased immune cell activation. Nevertheless, treatment with acetate promotes resolution of intestinal inflammation by GPR43 activation, thereby inducing apoptosis of inflammatory cells in colitis [22]. Acetate treatment has also been shown to reduce colonic inflammation in animal models by promoting Treg differentiation [88].

A recent study highlighted the important role of acetate production in preventing intestinal infection by its effect on the maintenance of gut epithelial barrier function [66]. Intriguingly, acetate may affect the production of reactive oxygen species (ROS) [22]. The production of ROS is involved in a wide spectrum of biochemical processes. The ability of ROS to activate an intracellular protein complex called the inflammasome is of crucial importance in IBD [54, 78, 79].

The role of the inflammasome in modulating the innate immune response in IBD is intimately related to the preservation of epithelial barrier integrity and the maintenance of gut homeostasis [50, 75]. Inflammasome complexes affect the innate immune response through activation by pathogen recognition NLRs [76]. NLRP6 and NLPR3 are key mediators of inflammasome complexes. NLRs activate caspase-1 and drive proteolytic processing of proinflammatory cytokines such as IL-1 and IL-18. These cytokines have evolved in intestinal epithelial cells to avoid overactive inflammatory responses against the host microbiota. Consequently, epithelial barrier integrity induces tissue repair following injury [65, 89, 90]. Several groups, using a common acute and chronic epithelial injury colitis mouse model based on the administration of DSS, reported an exacerbated disease severity in mice deficient in caspase-1, NLRP3, and NLPR6. These NLPRs are correlated with lower IL-1*β* and IL-18 production during colitis [89–92]. Interestingly, NLPR6-deficient mice have an altered gut microbiota (colitogenic bacteria), which together with the exacerbated colitis phenotype can be transferred to cohabitating WT mice. Therefore, NLRP6 participates in the steady-state regulation of the commensal microbiota and appears to be essential for preventing recurring colitis through the induction of basal secretion of IL-18 by epithelial cells [65]. Therefore, the inflammasome functions in the sensing of pathogens and the commensal microbiota by not only nonhematopoietic cells, such as the epithelial intestinal cells but also by hematopoietic cells [93]. Distinct inflammasome expression in different cell lineages may orchestrate different functions during mucosal inflammation. They cooperate to maintain host tolerance towards commensal microbes and to initiate a potent immune response towards pathogens in the gut [94]. Nevertheless, the factors inducing the formation of inflammasomes and the precise effector mechanisms for regulation of the microbiota and inflammatory response remain elusive. We do not yet know whether SCFAs or GPCRs influence inflammasome activation. However, the induction of ROS by SCFAs could be a new mechanism by which microbial components trigger inflammasome formation. Nevertheless, the inflammasome regulates innate immune responses by sensing endogenous and exogenous stimuli. Considering that the inflammasome induces essential inflammatory responses in IBD, the sensing of the microbiota by the inflammasome through the action of SCFAs could be a new protective mechanism associated with microbiota metabolites. Furthermore, microbiota metabolites can be considered analogous to microbe-associated molecular patterns (MAMPs), which signal through GPCRs to convey information about the microbiota and the host. These receptors provide molecular mechanisms associated with innate immunity that are involved in the recognition of MAMPs as well as the classical innate immune receptors such as TLRs and NLRs.

## 5. Obesity

Obesity has reached epic proportions, with incidence rates above 20% in most western countries [95]. It is characterized by abnormal or extensive fat accumulation that negatively affects health. Such conditions lead to reduced life expectancy and/or increased health complications such as heart disease, type 2 diabetes, obstructive sleep apnea, certain types of cancer, and osteoarthritis [96]. The development of obesity is a complex process involving primarily a combination of excessive food energy intake, lack of physical activity, and genetic susceptibility. A few cases, however, are caused by genes, endocrine disorders, slow metabolism, medications, or psychiatric illness [97]. The rise in incidence rates of obesity can be attributed to the Western diet [98]. An imbalance in the human gut microbiota has been associated with metabolic diseases including obesity, diabetes, and atherosclerosis [99]. Studies in both animals and humans have found fewer Bacteroidetes and more Firmicutes colonizing the gut [99].

The first evidence of the role of the gut microbiota in adiposity came from GF animal studies. Mice raised in a conventional environment had more total body fat in comparison to those raised under GF conditions. When GF mice were conventionalized, they experienced a dramatic increase in total body fat, and this increase was not associated with differences in food consumption or decreased energy expenditure [100]. The relation between gut microbiota and obesity was also verified in knockout and diet-induced obese mice. In such animal models, obesity was associated with changes in the composition and metabolic function of the microbiome [98]. Further evidence of the influence of the gut microbiota on obesity is provided by brain-gut axis studies. An increased intake of dietary fiber, which is fermented in the colon, has been reported to decrease body weight and glucose control. De Vadder and colleagues [101] have shown that SCFAs activate intestinal gluconeogenesis via a cAMP-dependent mechanism and a gut-brain neural circuit involving the fatty acid receptor FFAR3. Frost and colleagues [102] have demonstrated that colonic acetate crosses the blood-brain barrier and is taken up by the brain. SCFAs is also associated with activation of acetyl-CoA carboxylase and changes in the expression profiles of regulatory neuropeptides that favor appetite suppression.

**Figure 3:**
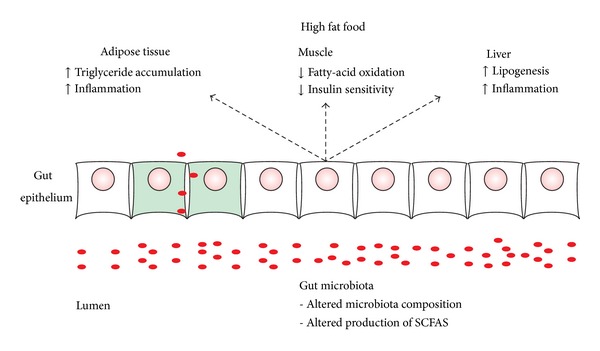
Effects of a high-fat diet. The altered microbial community of obese animals and humans promotes adiposity and decreased levels of short chain fatty acids and influences metabolic processes such as storage and metabolism of lipids in adipose tissue, muscle, and liver.

There are four main pathways that interfere with host energy storage. These pathways involve intestinal epithelial cells as sensors of microbial products and are believed to influence how the gut microbiome regulates host gene expression and affects energy expenditure and storage in the host [98, 103] (Figure 3). Colonization of GF mice with gut commensal bacteria alters the global intestinal transcriptional response and the cellular origins of selected responses by modulating the expression of genes involved in several important intestinal functions. These functions include nutrient absorption, mucosal barrier fortification, xenobiotic metabolism, angiogenesis, and postnatal intestinal maturation [103]. Studies using GF and conventionalized mice also revealed that the microbiota promotes the absorption of monosaccharides from the gut lumen, resulting in induction of* de novo* hepatic lipogenesis [104]. Fasting-induced adipocyte factor (FIAF), a circulating lipoprotein lipase inhibitor and member of the angiopoietin-like family of proteins, is selectively suppressed by conventionalism in the intestinal epithelium, liver, and adipose tissue of normal mice. Using GF, conventionalized, normal, and FIAF knockout mice, researchers established that FIAF suppression is essential for the microbiota-induced deposition of triglycerides in adipocytes. Their findings suggest that the gut microbiota is an important environmental factor that affects energy harvest from food and energy storage in the host.

A second pathway that affects host energy storage involves AMP-activated protein kinase (AMPK). AMPK is activated in response to metabolic stresses, and this activation results in an increased intracellular AMP to ATP ratio. Backhed and colleagues [105] reported that in contrast to mice with a gut microbiota, GF mice were protected against developing obesity after consuming a high-fat, sugar-rich Western diet. GF mice persistently remained lean despite a high caloric intake. This phenotype is associated with increased skeletal muscle and liver levels of phosphorylated AMPK, which stimulate fatty acid oxidation in peripheral tissues and lead to decreased glycogen content and increased insulin sensitivity in the liver [97]. These results suggest that the presence of a gut microbiota suppresses skeletal muscle fatty acid oxidation through a metabolic pathway that involves phosphorylation of AMPK. Moreover, GF knockout mice lacking FIAF were not protected from diet-induced obesity. GF FIAF−/−animals exhibited similar levels of phosphorylated AMPK compared to their wild-type littermates, but they had reduced expression of genes encoding for the peroxisomal proliferator-activated receptor coactivator Pgc-1*α* and enzymes involved in fatty acid oxidation. Based on these studies, GF mice are protected from diet-induced obesity by two independent but complementary mechanisms that result in increased fatty acid oxidation [98, 100, 105].

The host proteome has a limited number of glycoside hydrolases that are able to break down complex plant polysaccharides. The host microbiota synthesizes a large number of these enzymes, allowing them to break down complex carbohydrates into monosaccharides and SCFA. SCFAs diffuse passively and are recovered via monocarboxylic acid transporters, which also act as signaling molecules and ligands for GPR41, GPR43, and GPR109. SCFAs can be used as lipogenic substrates in host tissues but may promote fat storage via the activation of GPR41 and GPR43 receptors [26, 103, 106, 107]. Moreover, the activation of GPR43 by acetate and propionate contributes to the inhibition of lipolysis and adipocyte differentiation, thereby promoting the expansion of adipose tissue in animals fed a high-fat diet [108]. Because the capacity to ferment carbohydrates to SCFA varies among bacterial species (Bifidobacterium and* Bacteroides* species, e.g., are known to produce SCFAs), the actual composition of an individual's intestinal microbiota may play an important role in energy metabolism.

Finally, the low-grade inflammation and insulin resistance observed in obesity can be triggered by alteration of the gut barrier, leading to the higher plasma lipopolysaccharide (LPS) levels observed in obese individuals. Such conditions create a metabolic endotoxemia and drives obesity, insulin resistance, and systemic inflammation [108, 109].

## 6. Conclusions

Microbial signaling is required for immune development and homeostasis, whereas an intact immune system is necessary for maintenance of a healthy gut microbiota. Evidence presented herein suggests that some chronic inflammatory diseases are mediated or affected by the dysfunction of the gut microbiota and its metabolic products. Based on these observations, manipulation of intestinal microbiota may prevent or alleviate chronic inflammatory disease. The composition of the microbiota can be manipulated by antibiotics, probiotics, and dietary components. Probiotic consumption for the maintenance of a healthy gut has been practiced for over a century. In 1908, Elie Metchnikoff won the Nobel Prize for his discovery that ingestion of* Lactobacillus*-containing yogurt decreases the number of toxin-producing bacteria in the intestine. Several clinical and animal studies have suggested that probiotics and prebiotics can alleviate many inflammatory diseases such as asthma, obesity, and IBD. Clinical trials have indicated that feeding* L. rhamnosus* GG and* L. fermentum* to mothers during the prenatal and early postnatal periods may be effective in the treatment and prevention of early atopic disease in children. However, probiotics may not have the same positive effect on all subjects or on all chronic inflammatory diseases. One must also consider host dietary habits and probiotic actions such as production of SCFAs and direct DC activation. Additionally, many dietary components directly influence probiotic survival and activity. A high-fiber diet induces a healthy microbiota composition, leading to increased SCFA production, which has anti-inflammatory effects. Further studies are necessary to better understand the mechanisms by which probiotics improve chronic diseases. Additionally, probiotics could be genetically engineered to have desirable anti-inflammatory properties.
